# Association of Benzodiazepine Receptor Agonist Use With Changes in Psoriasis Severity in Adult Population: A Population-Based Study

**DOI:** 10.3389/fphar.2021.596375

**Published:** 2021-07-05

**Authors:** Ke-Ting Pan, I-Hsun Li, Hui-Han Kao, Yi-Hsien Chen, Pei-Xun Zhong, Li-Ting Kao

**Affiliations:** ^1^ Institute of Environmental Design and Engineering, Bartlett School, UCL, London, United Kingdom; ^2^ Graduate Institute of Aerospace and Undersea Medicine, National Defense Medical Centre, Taipei, Taiwan; ^3^ Department of Pharmacy Practice, Tri-Service General Hospital, Taipei, Taiwan; ^4^ School of Pharmacy, National Defense Medical Center, Taipei, Taiwan; ^5^ Department of Pharmacology, National Defense Medical Center, Taipei, Taiwan; ^6^ Graduate Institute of Life Sciences, National Defense Medical Center, Taipei, Taiwan; ^7^ Science and Technology Policy Research and Information Center, National Applied Research Laboratories, Taipei, Taiwan; ^8^ Department of Dermatology, Tri-Service General Hospital, National Defense Medical Center, Taipei, Taiwan; ^9^ School of Public Health, National Defense Medical Center, Taipei, Taiwan

**Keywords:** benzodiazepine receptor agonists, benzodiazepine, psoriasis, severity, safety

## Abstract

To date, it remains uncertain whether benzodiazepine receptor agonists (BZRAs) are aggravating factors even though these drugs can elevate the levels of biomarkers associated with the development of psoriasis. Therefore, this study aimed to investigate the association of BZRA use with changes in psoriasis severity. All data were sourced from the National Health Insurance system in Taiwan. We conducted a population-based retrospective cross-sectional study of 15,727 psoriasis patients who received BZRAs (BZRA users), and 18,856 psoriasis patients who did not receive BZRAs (nonusers). At least a 1-year washout period without any BZRA prescriptions was required. The main outcome was the change in psoriasis severity between before and after BZRA exposure. This study detected the exacerbation of psoriasis severity in mild psoriasis population by using a logistic model. Then, this study carried another logistic model among those patients who had severe psoriasis to calculate the odds ratios (ORs) for the improvement of the psoriasis severity. Among patients with mild psoriasis, BZRA users had a significantly higher probability of psoriasis severity exacerbation (IPTW-adjusted OR = 1.46). Mild psoriasis patients who received high and low doses of BZRAs had 1.70- and 1.39-fold higher probabilities of psoriasis severity exacerbation, respectively, than the non-users. Furthermore, in the severe psoriasis population, more low-dose BZRA users improved psoriasis severity than non-users. In conclusion, this study provided clinical evidence of the effects of BZRA use on patients with psoriasis severity. Among patients with mild psoriasis, high-dose BZRA users may be associated with the changes in psoriasis severity. However, low-dose BZRA exposure only slightly exacerbated disease severity among patients with mild psoriasis. Accordingly, clinicians should evaluate the risks and benefits of the BZRA usage.

## Introduction

Psoriasis is an immune-mediated chronic skin disease that affects 0.5–11.4% of the adult population worldwide (Boehncke and Schon, 2015; Michalek et al., 2017). This systemic inflammatory disorder is typically characterised by well-demarcated erythematous plaques with silvery scales (Lebwohl, 2003; Griffiths and Barker, 2007; Nestle et al., 2009). Additionally, this disease may worsen pain, disability and quality of life and place an economic burden on patients and the health care system (Kao et al., 2015; Ryan et al., 2015). Although psoriasis is not an immediately life-threatening disease, it is recognised to be associated with many comorbidities, including cardiovascular diseases, metabolic diseases and insomnia (Takeshita et al., 2017). Increasing evidence has also revealed that psoriasis-related pruritus and pain may be directly associated with insomnia (Gowda et al., 2010; Gupta et al., 2016).

Benzodiazepine receptor agonists (BZRAs) are frequently prescribed to treat insomnia and anxiety because they affect gamma-aminobutyric acid neurotransmission, thereby exhibiting anxiolytic and hypnotic properties (Olfson et al., 2015; Markota et al., 2016; Zhu et al., 2018). Although the use of BZRAs has increased in many countries and these drugs have emerged as a major challenge in health care, general practitioners and physicians may unavoidably prescribe BZRAs to some patients with psoriasis to improve their sleep quality (Donoghue and Lader, 2010). Nevertheless, increasing editorials and studies have suggested that BZRA can trigger or aggravate psoriasis (Fry and Baker, 2007; Basavaraj et al., 2010; Kim and Del Rosso, 2010). However, most prior studies could not demonstrate a causal relationship between BZRA use and changes in of psoriasis severity because of limitations of the study design (Cohen et al., 2005; Brauchli et al., 2009). Only one recent study found that BZRAs may be a risk factor for new-onset psoriasis (Li et al., 2019). To date, it is uncertain whether BZRAs are aggravating factors even though BZRAs can elevate some biomarkers associated with the development of psoriasis (Arican et al., 2005; Flisiak et al., 2010; Ku et al., 2018). No clear clinical evidence is available to guide physicians in the use of these drugs for patients with psoriasis. Therefore, this study aims to investigate the association between BZRA use and changes in psoriasis severity.

## Materials and Methods

### Data Source

All relevant data in this population-based study were sourced from the National Health Insurance (NHI) system in Taiwan. The dataset includes data for 2,000,000 individuals randomly selected from the 2005 NHI insured residents. Approximately 99% of residents are covered by this system, and 93% of clinics are contracted. In addition, this study was approved by the Tri-Service General Hospital Institutional Review Board (TSGHIRB No. 1-107-05–183), and the requirement for written informed consent was waived because the NHI database used in this study consisted of encrypted, secondary data released to the public for academic purposes.

### Study Sample Selection and Benzodiazepine Receptor Agonist Exposure Definition

**FIGURE 1:**
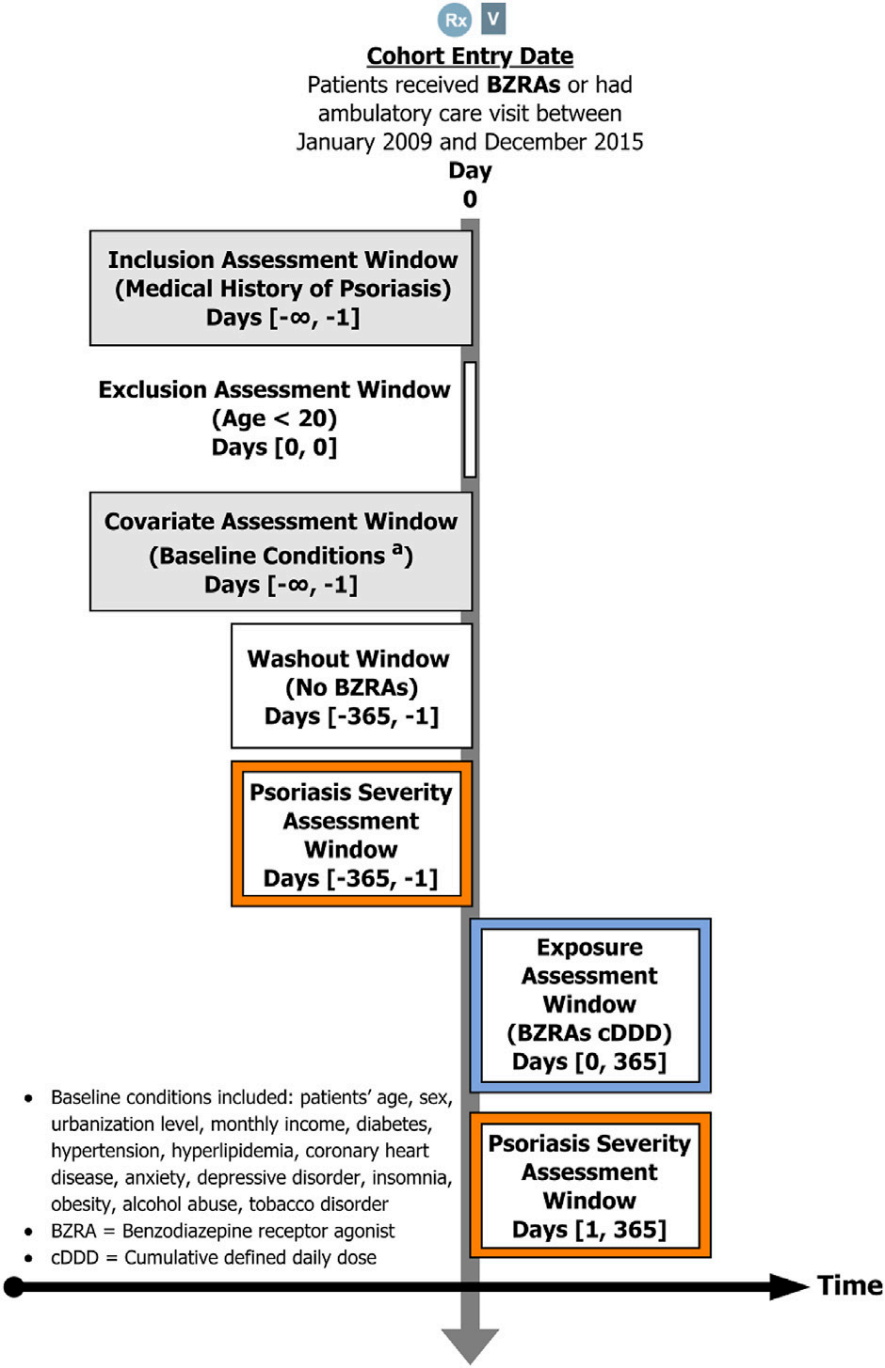
The design of the study.

We initially ensured that the source population had been diagnosed with psoriasis (ICD-9-CM codes 696) by at least one physician in the NHI program. This psoriasis definition was widely used in many previous studies and was considered for good diagnostic validity (Kao et al., 2015 and Li et al., 2019). Among the psoriasis population, we identified 17,268 BZRA users as the study group and 28,874 non-users as the comparison group using the period of January 2009 to December 2015. All the BZRAs in this study were prescribed by the physicians covered by NHI programme. Exposure to BZRAs, including BZDs and Z-drugs, was identified using Anatomical Therapeutic Chemical (ATC) codes. BZD anxiolytic and hypnotic agents were identified by ATC codes N05BA (benzodiazepine derivatives) and N05CD (benzodiazepine derivatives), respectively. Z-drugs (including zopiclone, zolpidem, zaleplon and eszopiclone) were identified by ATC code N05CF (benzodiazepine related drugs). To ensure incident BZRA treatment, at least a 1-year washout period without any BZRA prescriptions was required for all patients before them to be included in this study. The relevant strategy could eliminate the potential bias of BZRAs. This study further classified the BZRAs exposure strength by using cumulative defined daily dose (cDDD). BZRAs dose (cDDD) was evaluated as the sum of the dispensed defined daily dose of BZRAs during the study period (1 year). We then classified BZRAs users into two levels. High-dose BZRA users were patients who had received BZRAs for cDDD of ≥30 (Lan et al., 2015). The low-dose BZRA users were patients who had received the treatment for 1 to 29 cDDD during the study period. Then, we excluded patients who were loss to follow-up during the 1-year follow-up period (dead, emigration, or expatriate, etc.,). Patients younger than 20 years were also excluded to limit the study population to adults. Finally, this study defined 15,727 psoriasis patients who received BZRAs as the study group and 18,856 psoriasis patients who did not receive BZRAs as the comparison group. The date of the first outpatient visit for receiving BZRA prescriptions was identified as the entry date for BZRA users. Additionally, the date of a randomly selected outpatient visit during the period covered by the study was identified as the entry date for non-users. The design for the study is displayed in Figure 1.

This study further categorised the patients who had been diagnosed with psoriasis into two groups according to the last psoriasis treatment up to 1 year prior to entry date. Patients who had received any phototherapy or systemic medications (methotrexate, acitretin, cyclosporine and biological agents, including etanercept, adalimumab, ustekinumab and secukinumab) were deemed to have severe psoriasis (or patients receiving systemic treatment). The remaining psoriasis patients who had received topical therapies or did not receive any treatment were considered to have mild psoriasis (or patients receiving non-systemic treatment). This methodology of psoriasis severity categorisation was used and validated in many prior studies (Kao et al., 2015; Chiu et al., 2016; Chi et al., 2017).

### Outcome Measures

This study aimed to investigate the connection between BZRA use and changes in psoriasis severity. Thus, the main outcome was the change in psoriasis severity, which was indicated by a change in psoriasis treatment between before and after BZRA exposure (entry date) (Thorslund et al., 2013). Patients with mild psoriasis who received systemic treatments within 1 year after BZRA exposure were considered to have experienced an exacerbation of psoriasis severity. Moreover, patients with severe psoriasis who did not receive systemic treatments within 1 year after BZRA exposure were considered to have experienced an improvement of psoriasis severity.

### Measurement of Covariates

Several confounders might affect the association between BZRA use and subsequent changes in psoriasis severity. Therefore, sex, age, monthly income, urbanisation level of the residential area, and year of entry were all considered for each patient in this population-based study. Additionally, many potential determinants of the psoriasis severity, including hypertension (ICD-9-CM codes 401–405), hyperlipidaemia (ICD-9-CM codes 272.0–272.4), diabetes (ICD-9-CM codes 250), coronary heart disease (ICD-9-CM codes 410-414 or 429.2), insomnia (ICD-9-CM codes 780.5), depressive disorder (ICD-9-CM codes: 296.20–296.26, 296.30–296.36, 296.82, or 300.4), anxiety (ICD-9-CM code 300 except 300.4), alcohol abuse (ICD-9-CM codes 303), tobacco use disorder (ICD-9-CM 305.1, V1582, 989.84, or 649.0), and obesity (ICD-9-CM code 278), were also considered.

### Statistical Analysis

This study used the SAS (version 9.4) and GraphPad Prism 8. The standardised difference (SDiff) was carried out to evaluate the covariate balance, including patients’ demographic characteristics and comorbidities, between BZRA users and non-users. Additionally, logistic regression was used to evaluate the odds ratios (ORs) and 95% confidence intervals (CIs) for changes in psoriasis severity between before and after BZRA exposure. Specifically, in addition to relevant analyses based on original population, this study used the inverse probability of treatment weights (IPTW) strategy. IPTW is a propensity-score-based method that could efficiently and mathematically eliminate the baseline imbalance of measured demographics and comorbidities between BZRA users and non-users. Documented factors were used to evaluate the stabilised IPTWs in the study. The IPTW logistic models were employed to eliminate the bias of unweighted estimators. A two-tailed *p* value of <0.05 was used to evaluate statistical difference. This study further used restricted cubic spline function models with three knots in a multiple logistic regression model to evaluate the dose-response effects (Desquilbet and Mariotti, 2010).

## Results

Table 1 presents the baseline characteristics of the population, including an exposed (BZRA users) and unexposed group (nonusers). In the original population, the mean ages of the BZRA users and non-users were 45.8 ± 17.4 and 39.1 ± 16.1 years, respectively. The distributions of demographic characteristics and comorbidities were imbalanced between BZRA users and non-users.

**TABLE 1:** Demographic characteristics of the sampled patients at baseline.

Variable	Original population (*n* = 34,583)				
	BZRA users (*n* = 15,727)		Non-users (*n* = 18,856)		SDiff^a^
	No.	%	No.	%	
Age (years), mean (SD)	45.8 (17.4)		39.1 (16.1)		0.402
Sex	—	—	—	—	0.160
Female	6,784	43.1	9,630	51.1	—
Male	8,943	56.9	9,226	48.9	—
Psoriasis severity	—	—	—	—	0.058
Systemic treatment	448	2.9	371	2.0	—
Non-systemic treatment	15,279	97.2	18,485	98.0	—
Urbanisation level	—	—	—	—	0.102
1 (most urbanised)	5,453	34.7	7,139	37.9	—
2	4,694	29.9	5,692	30.2	—
3	2,836	18.0	3,400	18.0	—
4	2045	13.0	1974	10.5	—
5 (least urbanised)	699	4.4	651	3.5	—
Monthly income (NT$)^c^	—	—	—	—	0.135
0–18,000	2,956	18.8	3,081	16.3	—
18,001–35,999	8,247	52.4	9,204	48.8	—
≥36,000	4,524	28.8	6,571	34.9	—
Comorbidities	—	—	—	—	—
Hypertension	4,810	30.6	3,230	17.1	0.320
Hyperlipidaemia	4,740	30.1	3,447	18.3	0.280
Diabetes	3,021	19.2	2,244	11.9	0.203
Coronary heart disease	2,993	19.0	1795	9.5	0.274
Obesity	407	2.6	373	2.0	0.041
Tobacco use disorder	537	3.4	432	2.3	0.068
Insomnia	8,159	51.9	4,688	24.9	0.578
Depressive disorder	2,197	14.0	1,173	6.2	0.259
Anxiety	1,235	7.9	615	3.3	0.201
Alcohol abuse	222	1.4	100	0.5	0.090

**Note:** BZRA, benzodiazepine receptor agonist; SDiff, standardized difference.

a No covariates were considered to be imbalanced between groups if standardized difference (SDiff) < 0.1.

c The average exchange rate in 2010 was US$1.00 ≈ NT$30.

Table 2 displays the number of patients and risk of change in psoriasis severity between before and after BZRA exposure. Among patients with mild psoriasis, 153 patients (1.00%) experienced psoriasis severity exacerbation after BZRA exposure, compared with 110 (0.60%) non-users (crude OR = 1.69, adjusted OR = 1.56). Even after weighting using IPTW and adjustment for patients’ demographics and comorbidities (Model 4), BZRA users had a significantly higher probability of psoriasis severity exacerbation (IPTW-adjusted OR = 1.46, 95% CI = 1.15–1.86). Accordingly, this study observed that BZRA use may exacerbate psoriasis severity among patients with mild psoriasis. Conversely, among patients with severe psoriasis, 145 (32.37%) improved psoriasis severity after BZRA exposure, compared with 102 (27.49%) non-users. However, although a greater number of BZRA users with severe psoriasis improved severity than their counterparts who did not use the drugs, the OR for the change in psoriasis severity did not reach statistical significance.

**TABLE 2:** Number of patients and risk of psoriasis severity change between before and after BZRA exposure.

Baseline treatment	BZRA users (*n* = 15,727)	Non-users (*n* = 18,856)		Model 1^a^	Model 2^a^ ^,^ ^b^	Model 3^a^ ^,^ ^c^	Model 4^a^ ^,^ ^d^
	No. (%)		OR (95% CI)				
Patients receiving non-systemic treatment (mild psoriasis)	—	—		—	—	—	—
Psoriasis severity exacerbation (non-systemic → systemic)^e^	153 (1.00)	110 (0.60)		1.69*** (1.32–2.16)	1.56*** (1.20–2.02)	1.45** (1.14–1.84)	1.46** (1.15–1.86)
No severity change	15,126 (99.0)	18,375 (99.40)					
Total	15,279	18,485		--	--	--	--
Patients receiving systemic treatment (severe psoriasis)	—	—		—	—	—	—
Psoriasis severity improvement (systemic → non-systemic)^f^	145 (32.37)	102 (27.49)		1.26 (0.93–1.71)	1.22 (0.89–1.67)	1.05 (0.79–1.40)	1.04 (0.78–1.40)
No severity change	303 (67.63)	269 (72.51)					
Total	448	371		--	--	--	--

**Notes:** BZRA, benzodiazepine receptor agonist; OR, odds ratio; CI, confidence interval. *p ≤ 0.05, **p ≤ 0.01, ***p ≤ 0.001.

a Logistic regression was used to evaluate the ORs and 95% CIs.

b Adjusted for patients’ demographics and comorbidities.

c Weighted using the inverse probability of treatment weights (IPTW).

d Weighted using IPTW and adjusted for patients’ demographics and comorbidities.

e Exacerbate the severity of psoriasis.

f Improve the severity of psoriasis.

**FIGURE 2:**
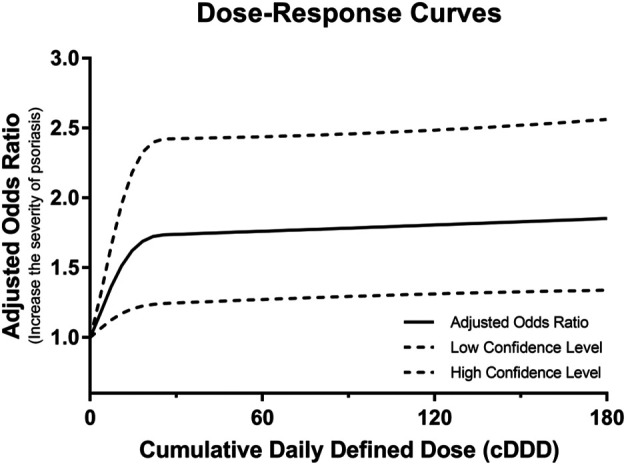
Dose-response curves for the adjusted odd ratios and 95% confidence intervals of the psoriasis exacerbation risk as a function of the benzodiazepine receptor agonist dose among patients with mild psoriasis.

Additionally, to assess whether the BZRA dose affected psoriasis severity, Table 3 presents the number of patients and risk of psoriasis severity change between before and after BZRA exposure according to the BZRA dose. Among patients with mild psoriasis, 1.32% of patients receiving high-dose BZRAs faced psoriasis severity exacerbation after BZRA exposure, compared with 0.60% of non-users with mild psoriasis (crude OR = 2.24). After weighting using IPTW and adjustment for confounders (Model 4), patients with mild psoriasis receiving high-dose of BZRAs had a 1.70-fold higher probability of psoriasis severity exacerbation. Moreover, the results illustrated that 0.90% of patients with mild psoriasis receiving low-dose BZRAs experienced psoriasis severity exacerbation after BZRA exposure, compared with 0.60% of non-users with mild psoriasis. After adjustment, patients with mild psoriasis receiving low-dose BZRAs had a higher possibility of psoriasis severity exacerbation (IPTW-adjusted OR = 1.39, 95% CI = 1.08–1.80). Hence, among patients with mild psoriasis, both low-and high-dose BZRA exposure may exacerbate the severity of psoriasis. We then conducted a dose-dependent analysis to determine whether the cumulative BZRA dose was associated with psoriasis exacerbation among the mild psoriasis population. As shown in Figure 2, an increased dose of BZRAs may significantly increase the probably of psoriasis severity exacerbation among patients with mild psoriasis. The aforementioned findings indicated that increasing the BZRA dose may exacerbate psoriasis among patients with mild psoriasis.

**TABLE 3:** Number of patients and risk of psoriasis severity change between before and after BZRA exposure according to the BZRA dose.

Baseline treatment	BZRA users (*n* = 15,727)	Non-users (*n* = 18,856)		Model 1^a^	Model 2^a^ ^,^ ^b^	Model 3^a^ ^,^ ^c^	Model 4^a^ ^,^ ^d^
	No. (%)		OR (95% CI)				
Patients receiving non-systemic treatment (mild psoriasis)	—	—		—	—	—	—
High-dose BZRA use	—	—		—	—	—	—
Psoriasis severity exacerbation (non-systemic → systemic)^e^	48 (1.32)	110 (0.60)		2.24*** (1.59–3.15)	1.76** (1.19–2.61)	2.02*** (1.42–2.87)	1.70** (1.19–2.45)
No severity change	3,584 (98.68)	18,375 (99.40)					
Total	3,632	18,485^g^		--	--	--	--
Low-dose BZRA use	—	—		—	—	—	—
Psoriasis severity exacerbation (non-systemic → systemic)^e^	105 (0.90)	110 (0.60)		1.52** (1.16–1.99)	1.48** (1.12–1.96)	1.32* (1.03–1.71)	1.39** (1.08–1.80)
No severity change	11,542 (99.10)	18,375 (99.40)					
Total	11,647	18,485^g^		--	--	--	--
Patients receiving systemic treatment (severe psoriasis)	—	—		—	—	—	—
High-dose BZRA use	—	—		—	—	—	—
Psoriasis severity improvement (systemic → non-systemic)^f^	40 (27.21)	102 (27.49)		0.99 (0.64–1.51)	0.99 (0.62–1.59)	0.78 (0.50–1.23)	0.73 (0.46–1.18)
No severity change	107 (72.79)	269 (72.51)					
Total	147	371^h^		--	--	--	--
Low-dose BZRA use	—	—		—	—	—	—
Psoriasis severity improvement (systemic → non- systemic)^f^	105 (34.88)	102 (27.49)		1.41 (1.02–1.96)	1.37 (0.97–1.93)	1.17 (0.86–1.59)	1.16 (0.84–1.59)
No severity change	196 (65.12)	269 (72.51)					
Total	301	371^h^		--	--	--	--

**Notes:** BZRA, benzodiazepine receptor agonist; OR, odds ratio; CI, confidence interval.

a Logistic regression was used to evaluate the ORs and 95% CIs.

b Adjusted for patients’ demographics and comorbidities.

c Weighted using the inverse probability of treatment weights (IPTW).

d Weighted using IPTW and adjusted for patients’ demographics and comorbidities.

e Exacerbate the severity of psoriasis.

f Improve the severity of psoriasis.

g Same patients

h Same patients. *p ≤ 0.05, **p ≤ 0.01, ***p ≤ 0.001.

This study also investigated the association between BZRA and the change in psoriasis severity among patients with severe psoriasis (Table 3). In this population, high-dose BZRA exposure was not associated with changes in psoriasis severity (27.21% for BZRAs users vs. 27.49% for non-users). However, the relevant findings indicated that 34.88% of patients with severe psoriasis receiving low-dose BZRAs experienced psoriasis severity improvement after exposure, vs. 27.49% of non-users with severe psoriasis. Although more low-dose BZRA users improved the psoriasis severity, there was no statistical association between low-dose BZRA use and changes in psoriasis severity among patients with severe psoriasis (IPTW-adjusted OR = 1.16, 95% CI = 0.84–1.59).

Because BZRA use can significantly increase risk of psoriasis exacerbation among patients with mild psoriasis, this study further estimated the potential risk of psoriasis severity exacerbation after BZRA exposure in different subgroups of patients with mild psoriasis. As presented in Table 4, BZRA exposure consistently exacerbated the severity of psoriasis in most subgroups among patients with mild psoriasis. Additionally, it was noteworthy that among patients with coincident diabetes, those with mild psoriasis who received BZRAs were 3.65-fold (95% CI = 1.98–6.73) more likely to face psoriasis severity exacerbation than their counterparts who did not use BZRAs even after weighting by using IPTW and adjustment for patients’ demographics and comorbidities.

**TABLE 4:** Number of patients and risk of psoriasis severity exacerbation between before and after BZRA exposure among different subgroups of patients with mild psoriasis.

Subgroups	Patients receiving non-systemic treatment (mild psoriasis)			Model 1^a^	Model 2^a^ ^,^ ^b^	Model 3^a^ ^,^ ^c^	Model 4^a^ ^,^ ^d^
	BZRA users	Non-users					
	No. (%)		OR (95% CI)			
Age group	—	—		—	—	—	—
Young population (<65 years old)	—	—		—	—	—	—
Psoriasis severity exacerbation (non-systemic → systemic)^e^	122 (0.95)	100 (0.59)		1.62*** (1.24–2.11)	1.46** (1.10–1.94)	1.43** (1.10–1.86)	1.39** (1.07–1.80)
No severity change	12,693 (99.05)	16,817 (99.41)					
Elderly population (≥65 years old)	—	—		—	—	—	—
Psoriasis severity exacerbation (non-systemic → systemic)^e^	31 (1.26)	10 (0.64)		1.99 (0.97–4.06)	1.88 (0.91–3.88)	1.61 (0.89–2.93)	1.48 (0.80–2.73)
No severity change	2,433 (98.74)	1,558 (99.36)					
Sex	—	—		—	—	—	—
Male population	—	—		—	—	—	—
Psoriasis severity exacerbation (non-systemic → systemic)^e^	65 (0.74)	34 (0.37)		1.99*** (1.32–3.02)	1.71* (1.11–2.63)	1.78** (1.18–2.68)	1.83** (1.21–2.75)
No severity change	8,704 (99.26)	9,080 (99.63)					
Female population	—	—		—	—	—	—
Psoriasis severity exacerbation (non-systemic → systemic)^e^	88 (1.35)	76 (0.81)		1.68*** (1.23–2.28)	1.48* (1.07–2.06)	1.31 (0.97–1.76)	1.30 (0.96–1.74)
No severity change	6,422 (98.65)	9,295 (99.19)					
Comorbidities	—	—		—	—	—	—
Diabetes population	—	—		—	—	—	—
Psoriasis severity exacerbation (non-systemic → systemic)^e^	50 (1.73)	10 (0.46)		3.78*** (1.91–7.47)	4.34*** (2.17–8.68)	4.09*** (2.23–7.50)	3.65*** (1.98–6.73)
No severity change	2,834 (98.27)	2,142 (99.54)					
Comorbidities	—	—	—	—	—	—
Non-diabetes population	—	—	—	—	—	—
Psoriasis severity exacerbation (non-systemic → systemic)^e^	103 (0.83)	100 (0.61)	1.36* (1.03–1.73)	1.16 (0.87–1.56)	1.12 (0.86–1.46)	1.11 (0.85–1.45)
No severity change	12,292 (99.17)	16,233 (99.39)			
Hyperlipidaemia population	—	—	—	—	—	—
Psoriasis severity exacerbation (non-systemic → systemic)^e^	57 (1.25)	26 (0.78)	1.60* (1.01–2.56)	1.67* (1.03–2.70)	1.59* (1.04–2.42)	1.56* (1.02–2.39)
No severity change	4,502 (98.75)	3,294 (99.22)				
Non-hyperlipidaemia population	—	—	—	—	—	—
Psoriasis severity exacerbation (non-systemic → systemic)^e^	96 (0.9)	84 (0.55)	1.62*** (1.21–2.18	1.48** (1.08–2.02)	1.39* (1.04–1.86)	1.40* (1.05–1.87)
No severity change	10,624 (99.1)	15,081 (99.45)				
Hypertension population	—	—	—	—	—	—
Psoriasis severity exacerbation (non-systemic → systemic)^e^	64 (1.39)	30 (0.97)	1.44 (0.93–2.23)	1.51 (0.97–2.36)	1.52* (1.01–2.28)	1.42 (0.94–2.15)
No severity change	4,538 (98.61)	3,071 (99.03)				
Non-hypertension population	—	—	—	—	—	—
Psoriasis severity exacerbation (non-systemic → systemic)^e^	89 (0.83)	80 (0.52)	1.61** (1.19–2.18)	1.50* (1.08–2.07)	1.42* (1.06–1.91)	1.39* (1.03–1.87)
No severity change	10,588 (99.17)	15,304 (99.48)				

**Notes:** BZRA, benzodiazepine receptor agonist; OR, odds ratio; CI, confidence interval. *p ≤ 0.05, **p ≤ 0.01, ***p ≤ 0.001.

a Logistic regression was used to evaluate the ORs and 95% CIs.

b Adjusted for patients’ demographics and comorbidities.

c Weighted using the inverse probability of treatment weights weight (IPTW).

d Weighted using IPTW and adjusted for patients’ demographics and comorbidities.

e Exacerbate the severity of psoriasis.

## Discussion

This population-based study investigated the connection between BZRA use and changes in psoriasis severity. Among the mild psoriasis population, this study observed that BZRA use may exacerbate psoriasis severity, and this positive association was consistently displayed in most subgroup analyses even after considering comorbidities and demographics. In addition, the findings revealed that high-dose BZRA use may significantly exacerbate the severity of psoriasis. However, low-dose BZRA exposure only slightly exacerbated disease severity among patients with mild psoriasis. Recently, many studies only focused on whether BZRAs can trigger new-onset psoriasis and it is unclear whether BZRAs can exacerbate the severity of psoriasis (Cohen et al., 2005; Brauchli et al., 2009; Kimball et al., 2012; Todberg et al., 2017; Li et al., 2019). As stated previously, most prior studies only focused on the relationship between BZRA use and psoriasis incidence, and they usually featured some methodological limitations, including the use of a case-control design, small sample sizes and the inclusion of patients aged <18 years (could not represent the adult population) (Cohen et al., 2005; Brauchli et al., 2009; Kimball et al., 2012; Todberg et alf., 2017). To date, no prior study investigated the connection between BZRA and psoriasis exacerbation.

Our study demonstrated that BZRAs may exacerbate disease severity among patients with mild psoriasis. The potential pathogenesis might be associated with the increased levels of some biomarkers. Many previous studies revealed that psoriasis is an inflammatory disease, and patients with this disease may have higher serum levels of cytokines, such as tumour necrosis factor (TNF)-α, interferon (IFN)-γ, (interleukin (IL)-6, IL-8, IL-12, IL-17 and IL-18 (Arican et al., 2005; Lowes et al., 2014; Baliwag et al., 2015). The relevant findings further indicated that high levels of IFN-γ, IL-12 and IL-18 may be associated with psoriasis severity (Arican et al., 2005). Recently, some studies reported that angiogenic factors may play an important role in the pathogenesis of psoriasis, and accumulating evidence indicates that the levels of vascular endothelial growth factor (VEGF) may be abnormal among the psoriasis population (Canavese et al., 2010; Flisiak et al., 2010; Henno et al., 2010; Wang et al., 2016; Sankar et al., 2017). In addition, one recent study observed that the levels of biomarkers, including IL-8, TGF-α and VEGF-A, were significantly elevated after 6 weeks of treatment with the BZRA lorazepam (Ku et al., 2018). Among overweight patients, the serum levels of IL-8, TGF-α, TNF-α, VEGF-A, VEGF-C and VEGF-D were significantly increased after lorazepam treatment (Ku et al., 2018). Accordingly, BZRAs may elevate the levels of some factors, such as IL-8, TNF-α VEGF, associated with the development of psoriasis (Arican et al., 2005; Flisiak et al., 2010; Ku et al., 2018). Therefore, it was plausible that BZRA may be an aggravating factor for psoriasis.

Our study also observed that a greater number of BZRA users, especially low-dose users, with severe psoriasis improved their severity than their counterparts who did not use BZRAs, although statistical significance was not reached. The relevant findings were interesting, and the potential aetiologies might be related to extremely poor sleep quality among patients with severe psoriasis. In general, patients with severe psoriasis may experience intolerable itch or pain that can further exacerbate disrupted sleep, chronic insomnia or poor sleep quality at night (Kaaz et al., 2019a; 2019b). Therefore, providing appropriate interventions to improve sleep quality among patients with psoriasis may improve the chronic inflammatory condition (Ganz, 2012). However, further studies are required to demonstrate the effects of BZRAs in patients with severe psoriasis.

This study had some unique strengths. First, it used data from a large population-based dataset. Therefore, the sample size and statistical power of this study were adequate to investigate the association between BZRA use and the change in psoriasis severity. In addition, the use of a population-based dataset eliminated selection bias, such as sampling bias, from the findings. Second, this study performed an IPTW strategy to demonstrate the actual association between BZRA and changes in psoriasis severity. This strategy reduced bias associated with differences in demographics and comorbidities among the study patients.

Nevertheless, several limitations should be mentioned for this study. First, the dataset provided no information regarding genetic factors, sunlight exposure, BMI, alcohol consumption or smoking habits, all of which represent risk factors for psoriasis. To eliminate the potential effects, our study used the obesity, tobacco-related disorders and alcohol abuse which recoded in the NHI database (ICD-9-CM codes) in place of BMI, alcohol or smoking habits and then considered these factors in the regression models. However, some residual bias may still remain and should carefully make inferences and conclusions. Secondly, the sample size of patients with severe psoriasis was relatively small. Most of the subjects identified in this study were patients with mild psoriasis. Thirdly, this study could not identify patients who illegally obtained BZRAs. These illegal BZRA users might have been classified as non-users, which would have led to misclassification bias in the study. Fourth, this study used the logistic models rather than time to event analysis and the cumulative dose of BZRAs could not be censored at the occurrence of the outcome. Further studies could be performed if they have collected relevant medical records. Finally, the information regarding the inflammatory biomarkers was unavailable in this study. Therefore, we could not estimate the influence of these factors in the relationship between BZRA exposure and psoriasis severity.

This study provided clinical evidence that BZRA use, especially high-dose exposure, may worsen disease severity among patients with mild psoriasis. However, low-dose BZRA exposure only slightly exacerbated psoriasis among patients with mild psoriasis and seems to have potential effects on slightly reducing disease severity among patients with severe psoriasis. Although the improvement of disease severity among patients with severe psoriasis was not significant, the findings in this study did provide a clinical insight into the association between BZRA use and changes in psoriasis severity. Clinicians should be alert to the potential effects of BZRAs and further evaluate the risks and benefits of the BZRA usage among psoriasis population. We recommend that if physicians prescribe BZRAs to patients with psoriasis to improve their sleep, a lower dose is recommended for patients with both mild and severe psoriasis. Further experimental studies on the effects of BZRAs on the psoriasis population are required and more epidemiological researches in other countries are still needed.

## Data Availability

The data analyzed in this study is subject to the following licenses/restrictions: Data sharing is not applicable to this article. Data used in this study are handled and stored by the Health and Welfare Data Science Center. Interested researchers can obtain the data through formal application to the Health and Welfare Data Science Center, Department of Statistics, Ministry of Health and Welfare, Taiwan (http://dep.mohw.gov.tw/DOS/np-2497-113.html).
